# Differential changes in gene expression in human neutrophils following TNF‐α stimulation: Up‐regulation of anti‐apoptotic proteins and down‐regulation of proteins involved in death receptor signaling

**DOI:** 10.1002/iid3.90

**Published:** 2015-12-02

**Authors:** Direkrit Chiewchengchol, Helen L. Wright, Huw B. Thomas, Connie W. Lam, Kate J. Roberts, Nattiya Hirankarn, Michael W. Beresford, Robert J. Moots, Steven W. Edwards

**Affiliations:** ^1^ Institutes of Integrative BiologyUniversity of LiverpoolLiverpoolUnited Kingdom; ^2^ Immunology Unit & Center of Excellence in Immunology and Immune‐mediated DiseaseDepartment of Microbiology, Faculty of Medicine, Chulalongkorn UniversityBangkokThailand; ^3^ Translational MedicineUniversity of LiverpoolLiverpoolUnited Kingdom; ^4^ Ageing and Chronic DiseaseUniversity of LiverpoolLiverpoolUnited Kingdom

**Keywords:** Death receptor signaling, neutrophils, TNF receptors

## Abstract

Responses of human neutrophils to TNF‐α are complex and multifactorial. Exposure of human neutrophils to TNF‐α in vitro primes the respiratory burst, delays apoptosis and induces the expression of several genes including chemokines, and TNF‐α itself. This study aimed to determine the impact of TNF‐α exposure on the expression of neutrophil genes and proteins that regulate apoptosis. Quantitative PCR and RNA‐Seq, identified changes in expression of several apoptosis regulating genes in response to TNF‐α exposure. Up‐regulated genes included TNF‐α itself, and several anti‐apoptotic genes, including BCL2A1, CFLAR (cFLIP) and TNFAIP3, whose mRNA levels increased above control values by between 4‐20 fold (*n* = 3, *P* < 0.05). In contrast, the expression of pro‐apoptotic genes, including CASP8, FADD and TNFRSF1A and TNFRSF1B, were significantly down‐regulated following TNF‐α treatment. These changes in mRNA levels were paralleled by decreases in protein levels of caspases 8 and 10, TRADD, FADD, TNFRSF1A and TNFRSF1B, and increased cFLIP protein levels, as detected by western blotting. These data indicate that when neutrophils are triggered by TNF‐α exposure, they undergo molecular changes in transcriptional expression to up‐regulate expression of specific anti‐apoptotic proteins and concomitantly decrease expression of specific proteins involved in death receptor signaling which will alter their function in TNF‐α rich environments.

## Introduction

TNF‐α is a pleiotropic cytokine that regulates the activity of many immune cells and tissues. It plays an important role in a number of physiological processes, such as cellular differentiation, but is also implicated in many pathological processes 1, 2, 3. Apart from its role in regulating acute and chronic inflammation, it is also involved in inflammatory diseases, such as septic shock, Crohn's Disease, systemic lupus erythematosus and rheumatoid arthritis 4, 5, 6, 7, 8.The central role of TNF‐α in many of these conditions is highlighted by the improvement in disease that often follows anti‐ TNF‐α therapy, notably in rheumatoid arthritis and juvenile idiopathic arthritis where timely anti‐TNF‐α therapy can lead to dramatic improvements in disease in many patients 9, 10, 11. Myeloid cells, including neutrophils, monocytes, macrophages and dendritic cells are recognized producers of TNF‐α. This cytokine can mediate its effects on cells by acting alone, or in combination with other cytokines, such as RANKL (Receptor Activator of Nuclear factor Kappa‐B Ligand) 12, 13.

The effects of TNF‐α on target cells are varied, and activation is mediated by the surface receptors, TNFR1 (TNFRSF1A, p55/p60) and TNFR2 (TNFRSF1B, p75/p80). Ligation of these receptors can lead to activation of a range of cellular responses, including cell survival, apoptosis, differentiation and proliferation 14, 15.The overall response of cells to TNF‐α is dependent upon the cell type, the concentration of TNF‐α, receptor binding and the intracellular signaling pathways that are activated. For example, TNF‐α signaling can lead to the generation of an apoptotic signal via FADD‐mediated activation of caspase 8, whereas TRADD/TRAF‐2 (TNF receptor associated factor‐2) activation can lead to activation of NF‐κB and triggering of downstream events that result in increased gene expression leading to an anti‐apoptotic or a proliferative response 16, 17, 18, 19, 20.

Neutrophils play a central role in TNF‐α signaling in immune activation during inflammation, by both generating TNF‐α and responding to it to become activated 21, 22, 23, 24, 25, 26, 27. Human neutrophils can be stimulated to express and release TNF‐α, and this cytokine is expressed on the cell surface of blood neutrophils of patients with uncontrolled rheumatoid arthritis 28. Moreover, the expression of surface TNF‐α on blood neutrophils decreases in line with disease improvement in patients who respond to anti‐TNF‐α therapy 28.

Neutrophil responses to TNF‐α are complex and varied in that low concentrations (∼10 ng/mL) can “prime” the respiratory burst, up‐regulate surface receptors, stimulate adhesion and delay apoptosis, whereas higher concentrations (>30 ng/mL) can promote apoptosis 27, 29. In view of these varied neutrophil responses triggered by TNF‐α stimulation, it is important to define responses to this molecule under conditions that mimic acute and chronic inflammation. On the one hand, neutrophils can express TNF‐α on their cell surface and release this molecule into the extracellular environment, thereby triggering events in adjacent cells. Alternatively, they themselves are stimulated by TNF‐α, into either anti‐ or pro‐apoptotic pathways, depending on the local concentration of this cytokine.

The aim of this study was to determine the molecular events that regulate neutrophil responses to TNF‐α signaling. We demonstrate that in response to low concentrations of TNF‐α, neutrophils are activated to express TNF‐α itself, exhibit delayed apoptosis and in parallel, down‐regulate expression of a number of genes and proteins that mediate death‐receptor signaling. These processes could therefore contribute to the fine tuning of responses of human neutrophils to TNF‐α in inflammatory conditions.

## Materials and Methods

### Reagents

The following reagents were used in this study: Polymorphprep (Axis‐Shield); RPMI 1640 media (Gibco, Paisley); Rapid Romanowsky stain (HD Supplies, Aylesbury, Bucks, UK); human AB serum and propidium iodide (Sigma, Gillingham, Dorset); TRIZOL, Superscript III First Strand cDNA Synthesis kit and FITC‐Annexin V (Invitrogen); RNAeasy kit, DNase and SYBR Green PCR kit (Qiagen); polyvinylidenedifluoride (PVDF) membranes and Immobilon Western Chemiluminescent HRP Substrate (Millipore). Primary antibodies (1:1,000) were from Cell Signaling while anti‐actin was from Abcam. Secondary antibodies (1:10,000) were anti‐mouse IgG (Sigma) and anti‐rabbit IgG (GE Healthcare).

### Isolation of neutrophils

This study was approved by the University of Liverpool Committee for Research Ethics. Neutrophils were isolated from heparinised peripheral blood of healthy donors using Polymorphprep, according to the manufacturers instructions. Red blood cell contamination was removed by hypotonic lysis. Neutrophils were re‐suspended in RPMI 1640 media to a concentration of 10^6^/mL. Neutrophil purity was assessed by staining with Rapid Romanowsky stain and was >97%. Re‐suspended neutrophils were incubated up to 6 h or overnight, with RPMI 1640 media at 37°C in a 5% CO_2_ incubator, with or without 10% (v/v) human AB serum, with and without TNF‐α, as indicated in the text.

### RNA isolation and real‐time PCR analysis

After incubation in the absence or presence of TNF‐α (10 ng/mL), neutrophils were pelleted, and RNA from the pellet was extracted with Trizol reagent using an RNAeasy kit, which included a DNA digestion step. Total RNA concentration and integrity was assessed using the Agilent 2100 Bioanalyser RNA Nano chip. cDNA was synthesized from 200 ng of total RNA from each sample, using the Superscript III First Strand cDNA Synthesis kit, in a 20 μL reaction volume, as per the manufacturer's instructions. Quantitative PCR analysis was carried out with the SYBR Green PCR kit, as per the manufacturer's instructions, using a Roche 480 LightCycler in a 96‐well plate using 1 μL of the above cDNA suspension in a 20 µL reaction volume. Target gene expression was quantified against a panel of housekeeping genes (GAPDH, β‐2 microglobulin, β‐actin, peptidylprolyl isomerase A). Initially, a Human Apoptosis RT^2^ Profiler PCR Array (SA Biosciences) was used to detect neutrophil transcripts whose levels were regulated by TNF‐α treatment. Thereafter, specific primers for genes of interest were purchased from PrimerDesign and Eurofins (Table 1). Levels of gene expression were normalized to the housekeeping genes by comparing Ct values, and data presented are normalized to GAPDH 30.

**Table 1:** PCR primer sequences.

Gene symbol	Product length	Sequence (5′‐3′)
TNF	219	f‐CAGAGGGCCTGTACCTCATC
		r‐GGAAGACCCCTCCCAGATAG
BCL2A1	82	f‐GAATAACACAGGAGAATGGATAAGG
		r‐CCAGCCAGATTTAGGTTCAAAC
CFLAR	93	f‐ATAGATGTGGTTCCACCTAATGTC
		r‐GTAGAGCAGTTCAGCCAAGTC
TNFAIP3	113	f‐AACATTTTGCTGCTGCCTCA
		r‐TCCTTCAAACATGGTGCTTCC
APAF1	94	f‐CTTCCAGCCAACCTATTTTCCT
		r‐CCTGATTAACCTTGGAGATAAAAGAA
BAX	101	f‐ATGGAGCTGCAGAGGATGAT
		r‐CAGTTGAAGTTGCCGTCAGA
CASP10	124	f‐GGAACGGACACACAACTCTC
		r‐AGCCCACTCACTTACAGACTAA
CASP8	95	f‐AGTAAGCAACAAGGATGACAAGA
		r‐ATCAATCAGAAGGGAAGACAAGTT
FADD	176	f‐CACAGACCACCTGCTTCTGA
		r‐CTGGACACGGTTCCAACTTT
FAS	104	f‐TGTAGTATGAATGTAATCAGTGTATGT
		r‐GATATTTCAGCAAAAGGTCATAGC
TNFRSF1A	88	f‐TGTGTCTCCTGTAGTAACTGTAAG
		r‐AGTCCTCAGTGCCCTTAACA
TNFRSF1B	145	f‐GTCCACACGATCCCAACAC
		r‐CACACCCACAATCAGTCCAA
TRADD	88	f‐GGTGCATCATTGGGGATTCT
		r‐GGGAGAAGGTGAGGCTGAT
GAPDH	106	f‐CTCAACGACCACTTTGTCAAGCTCA
		r‐GGTCTTACTCCTTGGAGGCCATGTG
ACTB	211	f‐CATCGAGCACGGCATCGTCA
		r‐TAGCACAGCCTGGACAGCAAC
B2M	114	f‐ACTGAATTCACCCCCACTGA
		r‐CCTCCATGATGCTGCTTACA
PPIA	60	f‐GCTTTGGGTCCAGGAATGG
		r‐GTTGTCCACAGTCAGCCATGGT

### RNA‐sequencing: Library generation and sequencing

Total RNA was enriched for mRNA using poly‐A selection, and standard Illumina protocols were used to generate 50bp single‐end read libraries, which were analyzed on a HiSeq 2000 instrument. Reads were mapped to the human genome (hg19) using TopHat v1.4.1 31, 32, and annotated using Cufflinks v1.3.0 32.Statistical analysis was carried out using Cuffdiff 33. The RNA‐Seq data discussed in this manuscript have been deposited in the NCBI's Gene Expression Omnibus (GEO) and are accessible through GEO Series accession number GSE40548 (http://www.ncbi.nlm.nih.gov/geo/query/acc.cgi?acc=GSE40548).

### Bioinformatics

Bioinformatics analysis was carried out using Ingenuity Pathway Analysis (Ingenuity® Systems, www.ingenuity.com).

### Western blotting

Isolated neutrophils were pelleted and washed with Phosphate Buffered Saline and lysed with boiling Laemmli lysis buffer at a concentration of 10^6^ cells/10 μL. Protein lysates for each sample were loaded onto a 10 % resolving polyacrylamide gels (SDS‐PAGE), and gel electrophoresis was run for 1 h. Separated proteins were transferred to polyvinylidenedifluoride (PVDF) membranes by electrophoresis for 80 min. The membranes were blocked with 5% non‐fat skimmed milk for 1h, incubated with primary antibodies and then incubated overnight with gentle agitation at 4°C. Primary antibodies were: rabbit anti‐human TNFR1 (TNFRSF1A), TNFR2 (TNFRSF1B), TRADD, FADD, Caspase‐10, FLIP (CFLAR) or mouse anti‐human Caspase‐8. After incubation with appropriate secondary antibodies, detection was by Immobilon Western Chemiluminescent HRP Substrate and careful exposure to film to avoid signal saturation. The membranes were then stripped for 30 min using stripping buffer (50 mM glycine, 150 nM NaCl, 0.1% (v/v) Tween‐20, HCl to pH 2.5), then re‐blocked and incubated with a monoclonal mouse anti‐actin antibody followed by anti‐mouse IgG secondary antibody. Band intensity was measured as an integrated density value, as determined by AQM Advance 6 software.

### Neutrophil apoptosis by flow cytometry

After incubation in the absence or presence of TNF‐α (10 ng/mL), neutrophils (2.5 × 10^4^) were stained with Annexin V‐FITC for 15 min (to measure early apoptosis) and then incubated with propidium iodide (1 µg/mL) (to detect changes in membrane permeability in late apoptotic cells). Stained cells were then analyzed on a Guava EasyCyte flow cytometer. Five thousand events were analyzed per sample.

### Statistical analyses

Statistical analyses were performed by using Student's *t*‐test. Data are shown as the mean ± SD, and the statistical significance of the differences between groups was determined. Differences with a *P*‐value of <0.05 were considered statistically significant.

## Results

### Effect of TNF‐α on neutrophil apoptosis and gene expression

Incubation of human neutrophils with 10 ng/mL of TNF‐α resulted in delayed apoptosis, as determined by decreased annexin V binding (due to surface phosphatidylserine exposure). Previous work has shown that this cell death is caspase dependent 34, 35. After 18 h incubation in the absence of TNF‐α, levels of apoptosis were 65% (± 4 %) while in TNF‐α‐treated cells, the level of apoptosis was significantly lower (53% ± 6%, *n* = 3, *P* < 0.05), confirming previous observations (Supplementary Figure S1) 36.

It has been previously reported that this concentration of TNF‐α also induces gene expression 23, 27 and so we initially used a commercial PCR array to identify genes involved in the regulation of apoptosis that were affected by this TNF‐α treatment. The PCR array that we used (see Materials and Methods) measured the relative expression of 84 key genes in the regulation of apoptosis (see http://www.sabiosciences.com/rt_pcr_product/HTML/PAHS-012Z.html for a full list of genes). Of these 84 genes, 13 were selected for further analysis (see list in Table 1) based either on their relative transcript abundance in neutrophils (Ct values of 20 or less) or on the basis that their relative levels were affected, positively or negatively, by TNF‐α treatment. Repeat experiments, on neutrophils isolated from different donors, as indicated in the figure legends, were then performed in individual quantitative PCR experiments using primers described in Table 1.

### Changes in expression of genes involved in death receptor signaling

**Figure 1:**
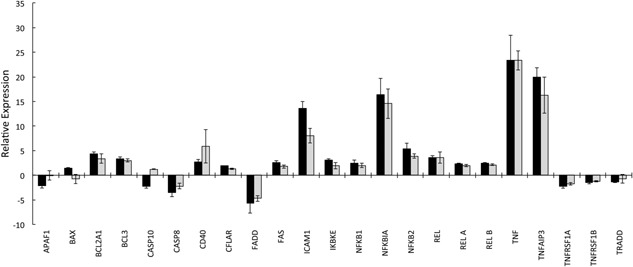
TNF‐α stimulation of neutrophil gene expression. Neutrophils were incubated for 1h in the absence (control) or presence of TNF‐α (10 ng/mL). Fold change in mRNA levels were quantified by real‐time PCR (normalized to GAPDH) (black bar) and RNA‐Seq (grey bar) (mean fold change ± SD, *n* = 3 for each dataset). Abbreviations: BCL2A1 (BFL‐1) = B‐cell lymphoma 2‐related protein A1; CFLAR (FLIP) = CASP8 and FAS‐like apoptosis regulator of FLICE‐like inhibitory protein; TNFAIP3 = Tumor necrosis factor α‐induced protein 3; Apaf‐1, Apoptotic protease activating factor 1; BAX, Bcl‐2‐associated X protein; CASP10, Caspase 10; CASP8, Caspase 8; FADD, Fas‐Associated protein with Death Domain; TNFRSF1A&1B (TNFR1&2), Tumor necrosis factor receptor superfamily member 1A&1B or Tumor necrosis factor receptor 1&2; TRADD, Tumor necrosis factor receptor type 1‐associated DEATH domain protein. For all data obtained by qPCR, the differences in expression levels following TNF‐α treatment were significantly different to control values (*P* < 0.05).

Figure 1 shows fold change in mRNA levels quantified by real‐time PCR (normalized to GAPDH) following the addition of TNF‐α (10 ng/mL) to neutrophils for 1 h. One of the genes whose expression was up‐regulated was TNF‐α itself, expression of which increased by over 20‐fold (Fig. 1). In addition, mRNA levels for TNFAIP3 (A20) increased by a similar magnitude following TNF‐α treatment. Transcripts for BCL2A1, CFLAR (FLIP), and FAS were also up‐regulated. However, expression of some genes involved in apoptosis control was significantly down‐regulated following TNF‐α treatment. These down‐regulated genes included APAF1, CASP8, CASP10, FADD, TNFRSF1A and TNFRSF1B.

In a parallel study, we used RNA‐Seq to measure the transcriptome of human neutrophils and identify changes induced by similar TNF‐α treatment 36. The qPCR results were therefore compared with results of these RNA‐Seq experiments, and both the qualitative and quantitative profiles of altered gene expression observed following TNF‐α treatment using these two independent methods, were very similar (Fig. 1).

**Figure 2:**
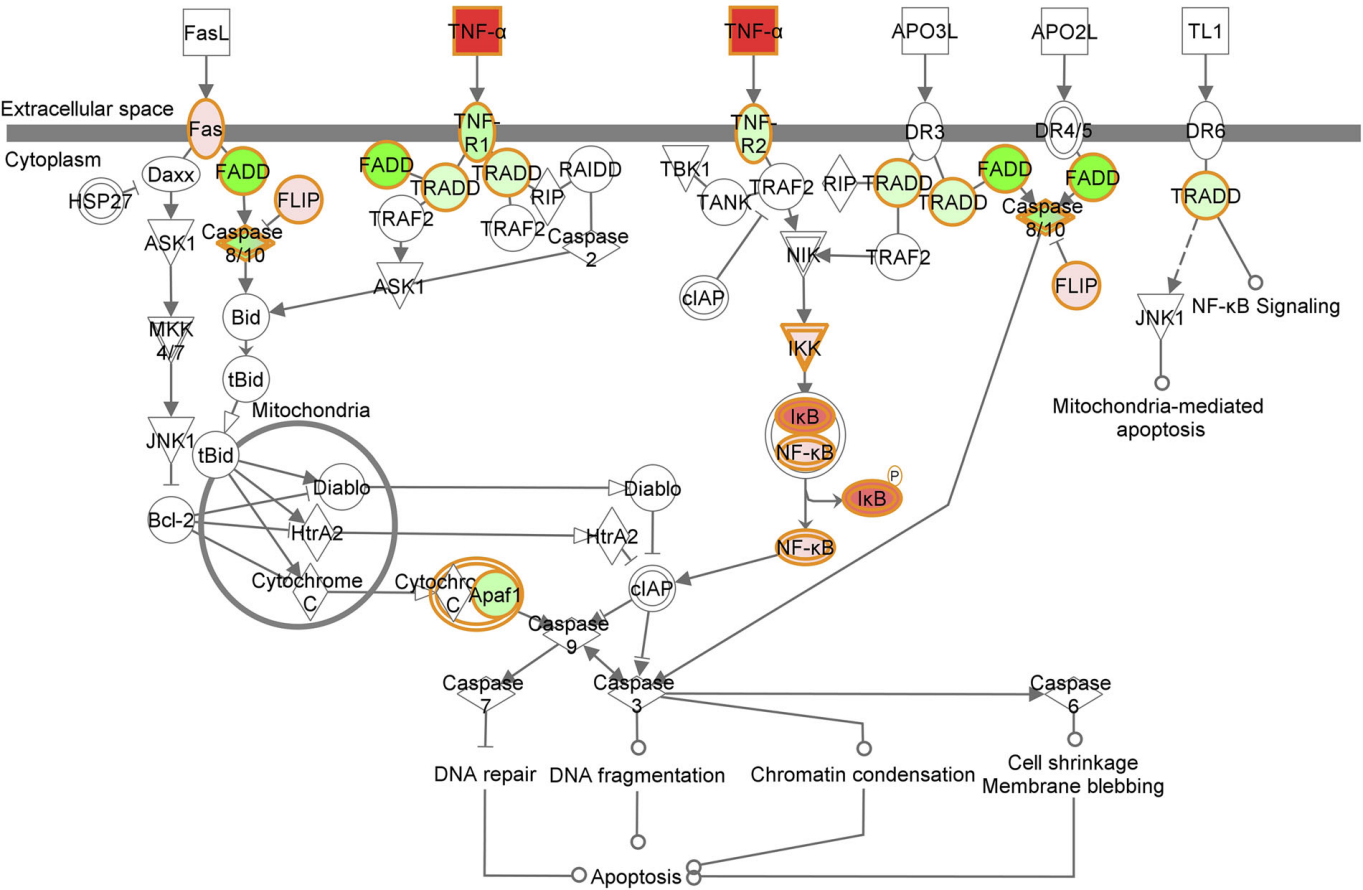
Bioinformatics analyses reveal changes in expression of genes associated with the Death Receptor Pathway. Data represent the mean fold change (*n* = 3) in expression following stimulation with TNF‐α for 1 h compared to untreated control measured using qPCR. Up‐regulated genes are shown in red and down‐regulated genes are shown in green, the analysis was generated using IPA (Ingenuity). Similar results were obtained when the RNA‐Seq data were analyzed in this way.

Bioinformatics analyses of the changes in gene expression following stimulation with TNF‐α for 1 h are shown in Figure 2. This scheme was generated by Ingenuity Pathway Analysis (IPA) software (Ingenuity^®^ Systems, http://www.ingenuity.com/) in which the fold change in gene expression values between the control (untreated) samples and the TNF‐α treated samples were projected onto the Death Receptor Signaling pathway. In this scheme, mRNA levels that decreased following TNF‐α treatment are shown in green, while those whose levels increased are shown in red. This scheme predicts that signaling of apoptosis via a range of death receptors would be down‐regulated as a result of the TNF‐α treatment of neutrophils.

### Time course of TNF‐α induced mRNA changes

**Figure 3:**
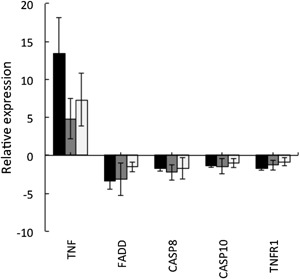
Changes TNF‐α‐activated gene expression are maximal after 1 h stimulation. Neutrophils were incubated for up to 6 h in the absence or presence of TNF‐α (10 ng/mL). Gene expression was analyzed by real‐time PCR (normalized to GAPDH). Samples were collected 1 h (black bar), 3 h (grey bar), and 6 h (open bar) after addition of TNF‐α. Values shown are mean (± SD) of three separate experiments. At 1 h time point, the changes in mRNA levels of all transcripts measured following TNF‐α treatment were significantly different to untreated values (*P* < 0.05).

The above transcriptome analyses were performed after 1 h incubation in the presence and absence of TNF‐α used at 10 ng/mL. In order to determine the kinetics of mRNA expression in response to TNF‐α stimulation, neutrophils were incubated for up to 6 h with TNF‐α and changes in gene expression were determined by qPCR. Maximal changes in gene expression were detected by 1 h incubation with TNF‐α, and extension of the incubation time to either 3 or 6 h did not result in any changes in expression of those genes of interest, above those observed after 1 h incubation (Fig. 3).

### Changes in protein levels following TNF‐α treatment

**Figure 4:**
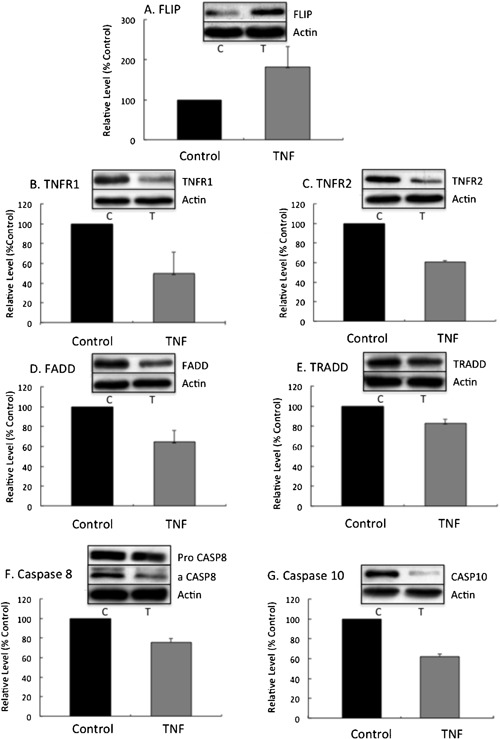
Changes in expression of pro‐ and anti‐apoptotic proteins after TNF‐α treatment. Neutrophils were incubated with TNF‐α (10 ng/mL) and at 2, 4, and 6 h incubation, protein extracts were made and subject to Western blotting. In each panel, representative Western blots of the protein of interest are shown, together with the corresponding actin control blot to ensure equivalence of protein load. Underneath each blot in each panel is summary data of three separate experiments showing mean values (± SD). For all data shown, there was a statistically significant difference between control (untreated) and TNF‐α‐treated neutrophils (*P* < 0.05). Panels A, D, and G are analyses after 4 h incubation with TNF‐α, while for the remaining panels, samples were analyzed after 6h incubation. For panel F, representative blots of pro‐capsase 8 (inactive) and cleaved (active) caspase 8 are shown, while quantitative data show the combined levels of pro‐ and cleaved‐caspase 8.

We then determined if these TNF‐α‐induced changes in mRNA levels were accompanied by corresponding changes in protein levels. Neutrophils were incubated for up to 6 h in the absence and presence of TNF‐α (10 ng/mL). No significant changes in protein levels were detected by Western blotting of protein lysates prepared following 2 h incubation (data not shown), but for most proteins under investigation maximal changes in expression were detected by 4–6 h incubation (data not shown). Figure 4 shows representative Western blot data and a summary of quantified data for FLIP, FADD and caspase 10 measured 4 h after TNF‐α treatment, while data for TNFR1, TNFR2, TRADD and caspase 8 are shown 6 h after TNF‐α treatment. In line with the qPCR/RNA‐Seq data, expression of FLIP protein was significantly enhanced following TNF‐α treatment (*P* < 0.05), while the expression of all other proteins investigated significantly decreased following TNF‐α treatment (*P* < 0.05). Thus, levels of expression of TNFR1, TNFR2, TRADD, and FADD were all significantly decreased after treatment. The antibody that we used in these studies detects both the pro‐ (inactive) and the cleaved (activated) forms of caspase 8 (Fig. 4F), and so we were able to quantify levels of both forms of this protein. Following TNF‐α treatment, levels of both the inactive pro‐form of the enzyme and the activated, cleaved form were decreased, indicating a down‐regulation of cellular protein levels that were independent of the level of apoptosis (which was decreased by TNF‐α exposure). The antibody that we used to detect caspase 10, only detected the uncleaved (inactive), pro‐form of the enzyme, which was decreased after TNF‐α treatment (Fig. 4G). As apoptosis was decreased in the presence of TNF‐α, this decrease in protein was likely to reflect decreased expression rather than increased cleavage.

## Discussion

This data presented in this study describe a potential new mechanism by which human neutrophils control their responsiveness to TNF‐α via up‐regulation of the expression of anti‐apoptotic genes and the simultaneous down‐regulation of expression of genes involved in death‐receptor signaling. Such a mechanism would be predicted to enable neutrophils to survive and maintain their functions within TNF‐α‐rich environments during inflammation. For example, in rheumatoid arthritis TNF‐α plays a central role in pathology and neutrophil function is activated in the blood of untreated patients 28. In such a disease, the mechanism described in this report would be predicted to affect the way in which neutrophils would respond to long term exposure to pathological levels of this cytokine. Down‐regulation of death receptor signaling would limit the responsiveness of neutrophils to TNF‐α, and hence limit their continued activation by this pro‐inflammatory cytokine. TNF‐α expression itself is markedly up‐regulated when neutrophils are exposed to TNF‐α, and so neutrophils can actively contribute to the TNF‐α signaling network that is observed in a number of inflammatory conditions (e.g. in rheumatoid arthritis) without the possibility of continuous, autocrine‐activation.

The effects of TNF‐α on human neutrophils are complex and varied, and are reported to be both concentration‐ and time‐dependent, as well as having differential effects on different sub‐populations of cells 26, 27, 29, 37, 38, 39, 40, 41, 42, 43. Previous reports have shown that TNF‐α down‐regulates neutrophil function by induction of shedding of TNFR1 and TNFR2 44, or internalization of TNFR1 45, which may partly explain de‐sensitisation of responsiveness during continuous exposure to this cytokine. Our data demonstrate that decreased *de novo* gene expression is also important in the modulation of neutrophil responses to TNF‐α, by down‐regulation of key proteins that are involved in TNF‐α mediated apoptosis. Such a mechanism may explain limitation of neutrophil responses to either exogenous or autocrine TNF‐α.

Most studies of neutrophil gene expression have, to date, focused upon identifying those genes whose expression is elevated or activated following inflammatory challenge. Few studies have reported decreased gene expression following activation, despite the fact that down‐regulation of key proteins can have a profound effect on neutrophil function and their subsequent contribution to infectious or inflammatory challenge. Here, we show significant down‐regulation of a number of genes that control responsiveness to death signals. The mechanisms responsible for decreased expression of particular genes are not known, but may involve activation of transcriptional repressors or chromatin re‐modelling, such as increased methylation or de‐acetylation of chromatin. While changes in mRNA levels were maximal 1 h after stimulation with TNF‐α, incubation periods in excess of 4 h were required before corresponding changes in protein levels were detected. This time delay between a decrease in mRNA and a decrease in protein levels, would allow an appropriate time window for the neutrophil to positively respond to the cytokine and then express new proteins, in this case anti‐apoptotic proteins, that alter cell function. There was some variability in the time after incubation with TNF‐α before the changes in protein levels were significantly different: some detectable by 4 h of incubation, others only detectable after 6 h incubation. This is possibly a consequence of differences in the turnover rates of these different proteins.

Using both quantitative PCR and RNA‐Seq to measure transcript levels in neutrophils following TNF‐α treatment, we demonstrated a very close correlation between the sets of data generated by these two independent methods. The former method is commonly‐used to measure expression levels of specific transcripts in human neutrophils, but the latter has not been used extensively to quantify the transcriptome of these cells. The benefits of RNA‐Seq over other transcriptome methods (qPCR, arrays) are significant 31, 32, and together with the ever‐decreasing costs of this technology, make this approach a cost‐effective way to study neutrophil function, both in vitro and ex vivo. One major difference in the results obtained by these two methods was in transcript levels for CASP10, which were decreased following TNF‐α treatment when measured by qPCR, but increased slightly when measured by RNA‐Seq. The reasons for this apparent difference are unknown, but this observation was consistently found in our PCR experiments (*n* = 6), that were performed with a different set of donors as those used for RNA‐Seq. Hence, this difference may represent donor variation.

This study demonstrated that several genes involved in NF‐κB signaling pathway were up‐regulated (e.g. NFKB1, NFKB2 and REL, as shown in Figs., 2 1) when neutrophils were exposed to TNF‐α. However, TNF‐α down‐regulates TNFR1 and TNFR2 receptors which would be predicted to down‐regulate TNF‐α mediated activation of this transcription factor. The NF‐κB signaling pathway can activate the transcription of many genes, including anti‐apoptotic genes such as Bfl‐1 (BCL2A1) in neutrophils, but it is activated by many other receptors such as TLRs, IL‐1R. Further analysis of our previously‐published RNA‐Seq data (*n* = 4) 36 (GSE40548) identified significant up regulation of IL‐1B receptor subunit‐2 and TLR2 by TNF‐α, whilst TLR1 was significantly down‐regulated (FDR < 0.01, Supplementary Figure S2A). Therefore, it is possible that while TNF‐α signaling may be down‐regulated by this mechanism, NF‐κB may still be activated via other inflammatory ligands. In the experiments described in this report, we have confirmed the increased expression of Bfl‐1 mRNA in response to TNF‐α, but were unable to detect increased expression of the protein due to lack of an antibody that reliably detects the human protein. Hence, we could not confirm increased Bfl‐1 protein expression following TNF‐α treatment, in spite of a large increase in mRNA levels. As requested by a reviewer, data for expression levels of a range of metalloproteinases are shown in Supplementary Figure S2B: expression of these genes was largely unaffected by TNF‐α treatment.

In summary, we have shown that neutrophils adapt to TNF‐α exposure by down‐regulating of expression of genes involved in the death‐receptor signaling, and this mechanism is likely to affect the ability of these cells to respond to this cytokine in inflammatory conditions. Such a mechanism will allow neutrophils to survive and function under TNF‐α ‐rich inflammatory conditions in vivo. Further work is now necessary to determine if this transcriptional re‐programming occurs in vivo in human diseases driven by TNF‐α (e.g. rheumatoid arthritis).

## Conflicts of Interest

There are no financial or commercial conflicts of interest.

## Supporting information

Additional supporting information may be found in the online version of this article at the publisher's web‐site

Figure Legends

**Figure S1**. TNF‐α delay of neutrophil apoptosis.

**Figure S2**. Previously published RNA‐Seq data deposited in the NCBI's Gene Expression Omnibus (GEO) and are accessible through GEO Series accession number GSE40548 (http://www.ncbi.nlm.nih.gov/geo/query/acc.cgi?acc=GSE40548), were analyzed for relative expression levels of the listed receptors (A) or metalloproteinases (B).
