# Supplementary material for: Differential changes in gene expression in human neutrophils following TNF‐α stimulation: Up‐regulation of anti‐apoptotic proteins and down‐regulation of proteins involved in death receptor signaling
Source: Immun Inflamm Dis. 2015 Dec 2;4(1):35–44. doi: 10.1002/iid3.90 (PMC4768069; doi:10.1002/iid3.90)
Supplement: Supplementary file 1 — Figure Legends [file IID3-4-35-s001.docx]

**Supplementary Figure legends**

**Supplementary Figure 1.** TNF-α delay of neutrophil apoptosis. Neutrophils were incubated for up to 18h in the absence or presence of TNF-α (10 ng/mL). Apoptosis was measured by flow cytometry using Annexin V-FITC and propidium iodide (PI) staining. Representative flow cytometry data showing decreased apoptosis (decreased annexin V binding) in TNF-α -treated neutrophils (B) compared to untreated cells (A). (C) shows quantitative data from repeat experiments shown in A and B. Values shown are means (± SD, n=3, * p<0.05, Student’s t-test).

Supplementary Figure 2. Previously published RNA-Seq data deposited in the NCBI’s Gene Expression Omnibus (GEO) and are accessible through GEO Series accession number GSE40548 (<http://www.ncbi.nlm.nih.gov/geo/query/acc.cgi?acc=GSE40548>), were analysed for relative expression levels of the listed receptors (A) or metalloproteinases (B). Untreated ( ) and TNF-α treated neutrophils ( , 10 ng/mL) were incubated for 1h before analysis of transcripts by RNS-Seq (as described in Methods and Materials). N= 3.
