# Supplementary figures and images for: Differential changes in gene expression in human neutrophils following TNF‐α stimulation: Up‐regulation of anti‐apoptotic proteins and down‐regulation of proteins involved in death receptor signaling
Source: Immun Inflamm Dis. 2015 Dec 2;4(1):35–44. doi: 10.1002/iid3.90 (PMC4768069; doi:10.1002/iid3.90)

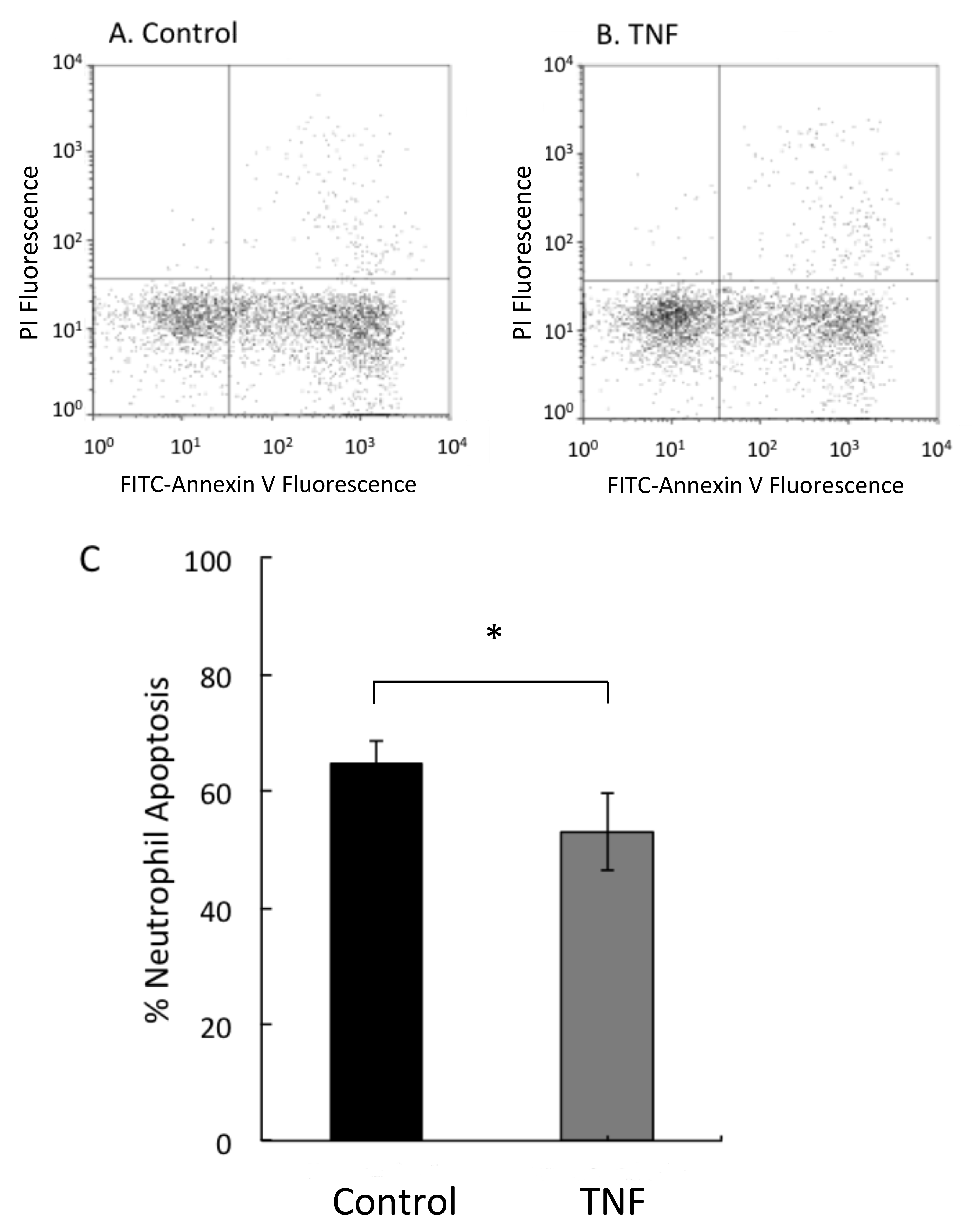

Supplement: Supplementary file 2 — Figure S1. TNF‐α delay of neutrophil apoptosis. [file IID3-4-35-s002.tif]

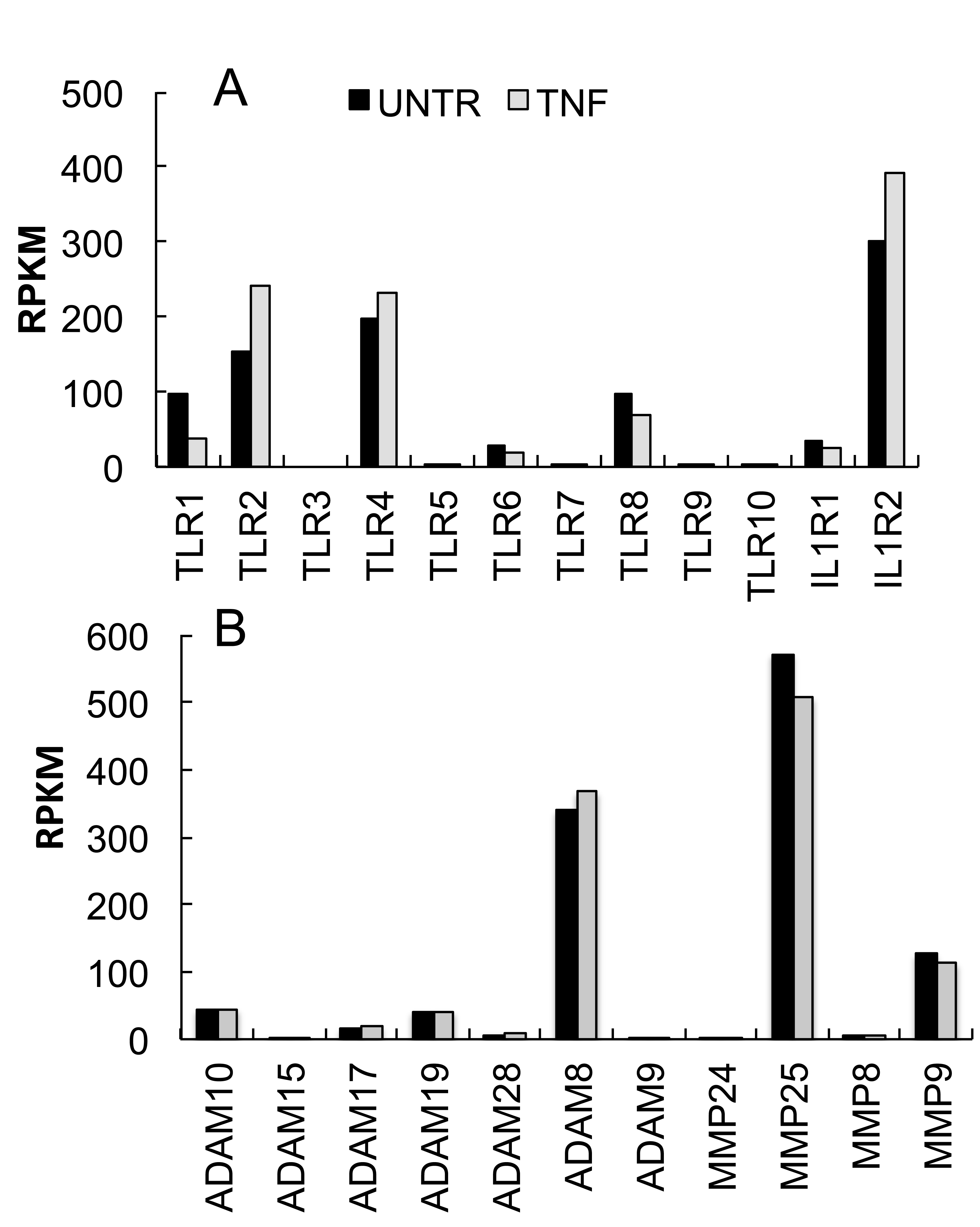

Supplement: Supplementary file 3 — Figure S2. Previously published RNA‐Seq data deposited in the NCBI's Gene Expression Omnibus (GEO) and are accessible through GEO Series accession number GSE40548 (http://www.ncbi.nlm.nih.gov/geo/query/acc.cgi?acc=GSE40548), were analyzed for relative expression levels of the listed receptors (A) or metalloproteinases (B). [file IID3-4-35-s003.tif]
